# Investigation of implantable signal transmission characteristics based on visible data of the human leg

**DOI:** 10.1186/s12938-017-0379-y

**Published:** 2017-07-04

**Authors:** Yue-Ming Gao, Yan-Ting Ye, Shi Lin, Željka Lučev Vasić, Mang-I Vai, Min Du, Mario Cifrek, Sio-Hang Pun

**Affiliations:** 1 College of Physics and Information Engineering, Fuzhou University, Fuzhou, 350116 China; 2 Key Lab of Medical Instrumentation & Pharmaceutical Technology of Fujian Province, Fuzhou, 350116 China; 3 Faculty of Electrical Engineering and Computing, University of Zagreb, Zagreb, Croatia; 4 State Key Laboratory of Analog and Mixed Signal VLSI, University of Macau, Macau, 999078 China; 5 Department of Electrical and Computer Engineering, Faculty of Science and Technology, University of Macau, Macau, 999078 China

**Keywords:** Channel modeling, Finite element model, Implantable signal transmission, Phantom model

## Abstract

**Background:**

Signal transmission characteristics between implanted medical devices and external equipment has been a common key issue, as has the problem of supplying energy to the devices. It can be used to enable signal transmission from implanted devices that the human body’s conductive properties. Using signal transmission by galvanic coupling is one of the most effective signal transmission methods.

**Methods:**

The signal transmission characteristics by galvanic coupling of implantable devices using a frequency range of 10 kHz to 1 MHz was analyzed in this article. A finite element (FEM) model and a phantom model established by visible human leg data were used to investigate the signal transmission characteristics of implant-to-surface, with implantable receiver electrodes at different locations.

**Results:**

The results showed that the FEM model and the phantom model had similar implantable signal transmission characteristics, with an increase of frequency, signal attenuation basically remained unchanged. The gain in signal attenuation in the fixed attenuation values fluctuated no more than 5 dB and signal attenuation values rose as the channel length increased.

**Conclusions:**

Our results of signal transmission characteristics of surface-to-implant will provide a theoretical basis for implantable transceiver design, and for realization of a recharging method for implanted medical devices.

## Background

Implanted medical devices enable long-term continuous monitoring of physiological changes, diagnosis and analysis of some chronic diseases, and control of organ feature loss. They also enable more comprehensive health monitoring [1–4]. Signal transmission between those devices and external equipment has been a common key issue, as has the problem of supplying energy to the devices. It can be used to enable signal transmission from implanted devices that the human body’s conductive properties. This is one of the most effective signal transmission methods that using signals transmission by galvanic coupling, because it does not have to design the antenna of the transceiver, yet the power consumption maybe further decrease. Moreover, the surface-to-implant recharging of implanted medical devices become possible [5], since the path loss of the signal transmission is not very high.

Several research groups have investigated signal transmission from implantable devices in the human body. Lindsey et al. [6] implanted a sensor in a cadaver knee joint and measured the tension of the ligaments from the surface of the leg. Experimental results show that for signal transmission from implant to surface, signal attenuation is closely related to the amount of current, the size of the surface electrode, and the distance between the electrodes. Wegmueller et al. [7] designed a system that could achieve signal transmission from implant to implant. They experimented with the electrical properties of emulated muscle. Shiba et al. studied signal transmission from human tissue to the body surface, verifying the feasibility of implant-to-surface signal transmission [8]. Ferguson et al. [9] described and compared several methods of signal transmission using the conductive properties of the body between implanted medical devices. In terms of energy transmission, [10, 11] analyzed the feasibility of using volume conduction for energy transfer. A transcutaneous battery recharging circuit unit was designed that takes advantage of skin volume conduction. On that basis, a low-power digital communication model based on volume conduction was presented.

Most of the aforementioned studies of implant signal transmission using the conductive properties of the body were carried out in physiological saline or other liquid that had the electrical properties of human tissues. However, that approach cannot emulate the complex anatomical and geometrical characteristics of the human body, and leads to experimental results not completely consistent with those in realistic. Most research has concentrated on verifying the feasibility of implant signal transmission; research on the characteristics of the transmission is scarce. We investigated those characteristics to better understand effective signal transmission and to offer a theoretical analysis of the recharging of implanted medical devices. In this research, surface-to-implant signal transmission characteristics in a frequency range from 10 kHz to 1 MHz was analyzed using a finite element method (FEM) model and a phantom model. We established an implantable FEM model using visible human leg data and FEM method. We then synthesized a material whose dielectric properties were similar to those of human tissues. An implantable phantom model was made using the material and a visible human leg outer contour. Signal transmission characteristics from the surface to the implant were analyzed by doing implant experiments in a FEM model and a phantom model. The mutual complementation and authentication of the FEM and phantom models lent veracity and reliability to the implantable signal transmission characteristics studied in this article. Our results will provide a theoretical basis for implantable transceiver design, and for realization of a recharging method for implanted medical devices.

## Methods

### The implantable leg FEM model

In the human legs, the tissues of fat, muscle and bone accounted for a larger volume, so that the influence of fat, muscle and bone on signal transmission cannot be ignored. And the skin is directly contacted with the electrode, the ion current and the electron current can be exchanged on the surface of the skin-electrode [12]. It plays an important role in the signal transmission process of surface-to-implant. Therefore, in this article, neglecting other small tissues such as blood vessels, lymph, mucous membrane, the human leg model was divided into four layers of skin, fat, muscle and bone [13].

Thus, a four-layer leg model was reconstructedusing transverse anatomical fault images of a male provided by the U.S. National Library of Medicine and the University of Colorado. First, the leg structure is divided into several different tissues, including skin, fat, muscle and bone. Next, the outline of each tissue layers on every anatomical image are extracted automatically, and the contour line of tissues are reconstructed through three-dimensional reconstruction software GEOMAGIC Studio (GEOMAGIC Inc., North Carolina, USA). Finally, using SOLIDWORKS (Dassault Systemes S.A., Massachusetts, USA) to fill each layer with the respective tissues to acquire a personalized leg model [14].

**Fig. 1:**
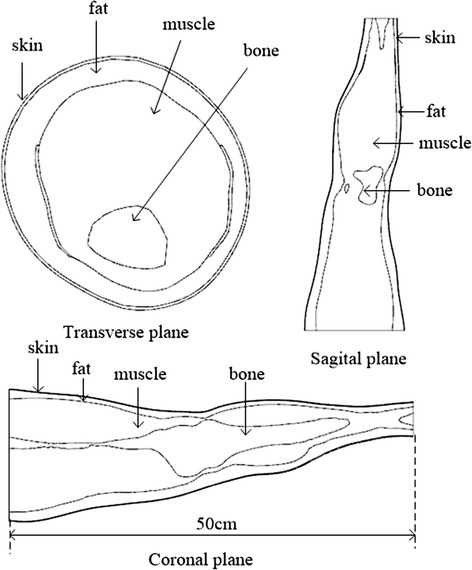
Three views of the human body leg model

The four-layer leg model included skin, fat, muscle and bone layers, as shown in Fig. 1. The complex frequency behavior of the dielectric properties of human tissue; that is, conductivity σ and permittivity ε, were derived from the parametric modes of Gabriel [15]. The FEM simulation is conducted in the COMSOL MULTIPHYSICS 5.2 (COMSOL Inc., Stockholm, Sweden).

**Fig. 2:**
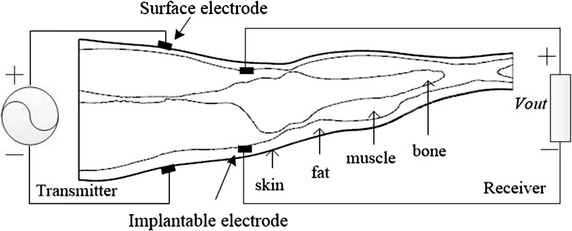
Implantable signal transmission system

In an implantable signal transmission system (Fig. 2), a pair of transmitter electrodes was attached to the skin surface so the voltage or current signal could pass into the body [16]. A pair of implantable receiver electrodes was attached at the junction of the muscle and fat layers to receive electrical signals. The electrode dimensions were 4 cm × 4 cm, 1 mm thick, with a conductivity of 6 × 103 S/m and a relative dielectric constant of 1.

**Fig. 3:**
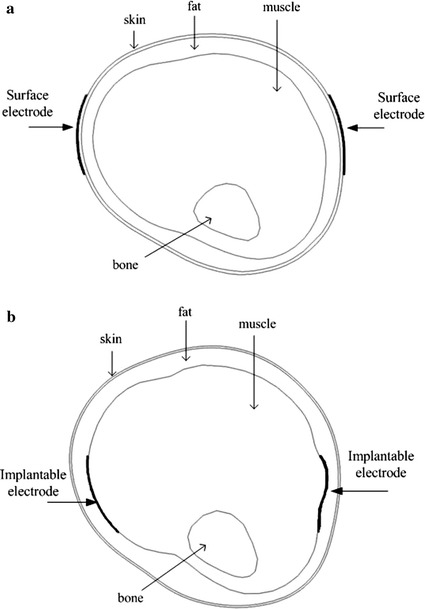
The transverses plane of electrode. **a** Surface transmitter electrodes. **b** Implantable receiver electrodes

To ensure close contact of the electrodes with human tissue, we positioned the surface transmitter electrodes and implantable receiver electrodes in the following ways: The contour of the skin layer to the external upset was 1 mm and made a Boolean intersection with a rectangular parallelepiped whose cross section was 4 cm × 4 cm, to obtain a transmitter electrode that was in close contact with the skin surface. Voltage or current passed into the body through the transmitter electrodes. Similarly, the contour of the muscle layer to the external upset was 1 mm and made a Boolean intersection with a rectangular parallelepiped whose cross section was 4 cm × 4 cm, to obtain an implantable receiver electrode that was in close contact with the muscle layer. The surface transmitter electrodes and implantable receiver electrodes shown in Fig. 3.

Based on the geometry model of the human leg (Fig. 1), the mechanism of the implantable signal transmission was investigated at modes A, B, and C, as described in “Simulation mode setups”. Because the total charge density in a human body is zero, a Maxwell equation could be equivalent to the Laplace equation [17–19].

1 $$- \nabla \times \left[ {\left( {\sigma + i\omega\upvarepsilon_{0} \varepsilon_{i} } \right)\nabla V} \right] = 0$$

where *σ* is the electrical conductivity, ω is the angular frequency, *ɛ*_0_ is the permittivity of free space, *ɛ*_i_ is the permittivity of the medium, and *V* is the potential difference of the body surface.

The input electrical signal at the surface transmitter electrodes was

2 $$V = V_{0}$$

where *V*_0_ was the amplitude of the voltage injected into the limb.

Current and voltage continuity conditions of the receiving electrodes and of the surface of the human model were given by:

3 $$\left\{ \begin{aligned} V_{l - 1} = V_{l} \hfill \\ J_{l - 1} = J_{l} \hfill \\ \end{aligned} \right.$$

where, $J_{l - 1}$, $J_{l}$ and $V_{l - 1}$, $V_{l}$ indicate the current density and the voltage of the adjacent tissues, and l indicates the tissue layer.

Thus, a default fine free tetrahedral element is used to mesh the human body leg model. The solution for the model is obtained by running the direct solver PARDISO in a fully coupled manner.

### Simulation mode setups

In accordance with the location of the implantable receiver electrodes, simulation devices were divided into the following three modes:

#### Mode A

**Fig. 4:**
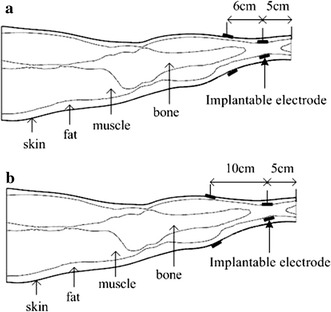
*Mode A:* Implantable receiver electrode placement at the calf. **a** Transmitter and receiver electrodes separated by 6 cm. **b** Electrodes separated by 10 cm

As shown in Fig. 4, the receiver electrodes were implanted in the calf 5 cm from the bottom of the leg model at the junction of the fat and muscle layers. The transmitter electrodes were placed on the surface of the skin layer. Transmitter and receiver electrodes were separated by 6 and 10 cm.

#### Mode B

**Fig. 5:**
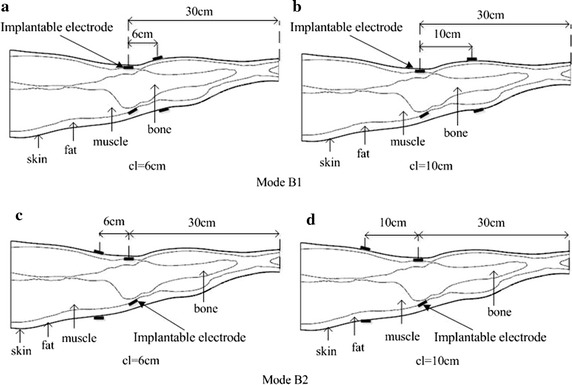
*Mode B:* Implantable receiver electrodes placement near the knee of the leg model. Mode B1, in the direction of the calf, **a** 6 cm between electrodes, **b** 10 cm between electrodes. Mode B2, in the direction of the thigh, **c** 6 cm between electrodes, **d** 10 cm between electrodes

As shown in Fig. 5, the receiver electrodes were implanted near the knee 30 cm from the bottom of the leg model at the junction of the fat and muscle layers. The transmitter electrodes were placed on the surface of the skin layer. Mode B1: Transmitter and receiver electrodes were separated by 6 cm (Fig. 5a) and 10 cm (Fig. 5b) toward the direction of the calf. Mode B2: Transmitter and receiver electrodes were separated by 6 cm (Fig. 5c) and 10 cm (Fig. 5d) toward the direction of the thigh.

#### Mode C

**Fig. 6:**
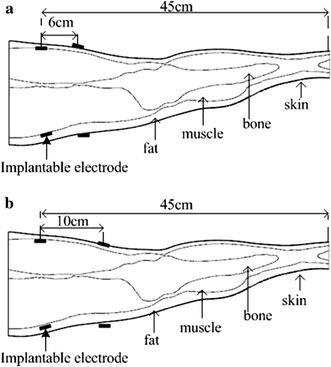
*Mode C:* Implantable receiver electrode placement at the thigh. **a** 6 cm between electrodes. **b** 10 cm between electrodes

As shown in Fig. 6, the receiver electrodes were implanted at the thigh 45 cm from the bottom of the leg model at the junction of fat and muscle layers. The transmitter electrodes were placed on the surface of the skin layer. Transmitter and receiver electrodes were separated by 6 cm and 10 cm.

### The implantable leg phantom model

The FEM fully takes into account the characteristics of human anatomy and can describe the distribution of electromagnetic fields in the organism, but its shortage was not suitable for the actual performance test. An implant phantom model consists of synthetic material whose dielectric properties are similar to those of human tissues. Using this model to emulate the human body enables more realistic experimentation. The FEM and phantom models we used enabled research of the channel characteristics of implantable signal transmission to be more realistic, accurate, and reliable.

The greatest influence on signal transmission through the human body by galvanic coupling are the skin, fat, and muscle layers [19, 20]. The effects of the periosteum and bone marrow are minor [15], so the influence of bones can be ignored. As shown in Table 1, it can be finding that the conductivity of the fat and skin layers is almost the same, when the frequency is near 40 kHz [16], so they are equivalent to a single layer. The phantom model was divided into muscle and skin–fat layers.

**Table 1:** Conductivity of each human body layer at different frequencies

Conductivity (S/m)	Frequency (Hz)			
	10,000	40,000	70,000	100,000
Muscle	0.34083	0.34977	0.35579	0.36185
Fat	0.00293	0.02167	0.04475	0.06583
Wet skin	0.02383	0.02419	0.02433	0.02441

**Fig. 7:**
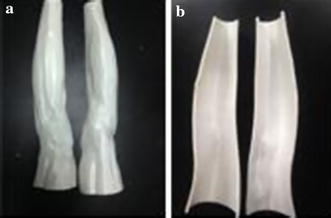
3D-printed outer contour molds of human legs. **a** Outer contour mold of muscle layer (*outside layer*). **b** Outer contour mold of skin–fat layer (*inside layer*)

The human leg contour model was rebuilt using visible human leg data, and then the outer contour molds of the muscle and skin–fat layers were printed by a 3D printer, as shown in Fig. 7.

Agar, potassium chloride, hydroxyethyl cellulose (HEC) and distilled water were mixed as shown in Table 2 to emulate human tissue. The electrical conductivity of the leg model was changed by adjusting the quantity of potassium chloride to approximate the conductivities of muscle and skin–fat layers.

**Table 2:** Proportions of ingredients to emulate human muscle and skin–fat layers

Tissue	Ingredient				
	Agar (g)	KCl (g)	HEC (g)	Distilled water (mL)	Methylene blue
Skin–fat	40	0.1652	80	1400	Appropriate
Muscle	40	2.8	80	1400	None

**Fig. 8:**
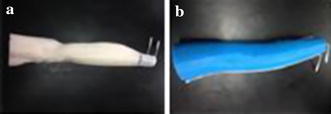
Phantom model of mode A. **a** Muscle layer. **b** Complete model of mode A showing outer skin–fat layer

After the muscle layer of the phantom model was produced, we put receiver electrodes in fixed positions at the surface of the layer according to modes A, B, and C. Then we covered the muscle layer with the outer skin–fat layer. During the latter’s production we added a biological stain called methylene blue to distinguish the two layers (Fig. 8).

**Fig. 9:**
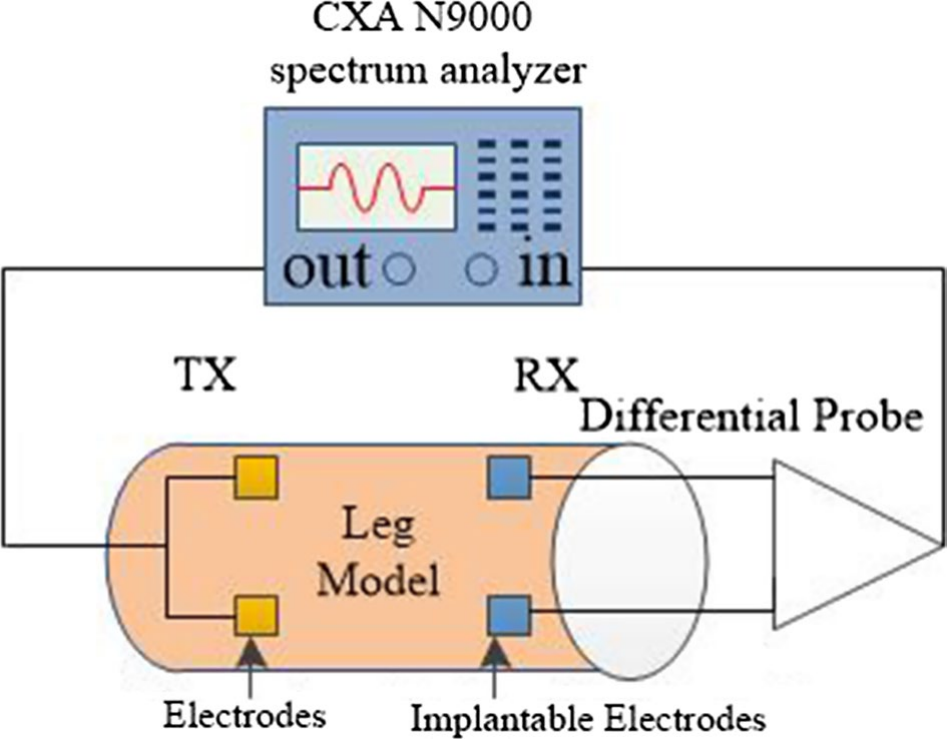
Galvanic coupling implantable signal transmission experiment in the phantom model

Figure 9 is a block diagram of the galvanic coupling implantable signal transmission experiment in the leg phantom model. A CXA Agilent N9000 spectrum analyzer was used in the experiment of galvanic coupling implantable signal transmission in the phantom model. A 0 dBm output signal from the extremity of the TX was injected into the leg model through the transmitting electrodes. The receiving signal was detected by a differential probe in order to avoid the results being affected by the human body and the ground connecting to form a looping-in, and then voltage gain was shown on the screen.

The voltage gain was used to represent the channel attenuation characteristics, with their expression shown in Eq. (4):

4 $$G{\text{ain}}({\text{d}}B) = 20 \cdot \log (V_{\text{r}} /V_{\text{t}} )$$

where Gain are the channel attenuation characteristics in dB, *V*_r_ is the receiving voltage, and *V*_*t*_ is the transmitting voltage.

## Results

As described in “Methods”, the simulation devices had three modes: A, B, and C. The channel characteristics of the implantable signal transmission of the FEM and phantom models were investigated at each of the modes.

**Fig. 10:**
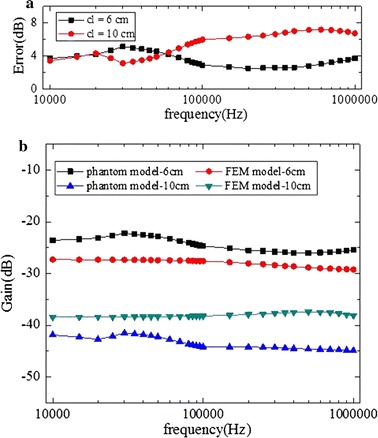
In mode A, a comparison between implantable FEM model simulation results and phantom model experiment results. **a** Error of the phantom and FEM models. **b** Results of the phantom model experiment and the FEM model simulation

Figure 10 shows the results when the implantable receiver electrodes were placed in the calf (mode A). The FEM model signal variation was consistent with that of the phantom model, with little change in the increase of frequency. The gain in a fixed attenuation value fluctuation was no more than 5 dB. Figure 10 also shows that there was higher attenuation values with the channel length increased. For either channel length (cl = 6 cm and cl = 10 cm), the errors between the FEM model and the phantom model were limited to 8 dB. When cl = 6 cm, the phantom model signal gain was less than that of the FEM model, and when cl = 10 cm, the FEM model signal attenuation was less than that of the phantom model

**Fig. 11:**
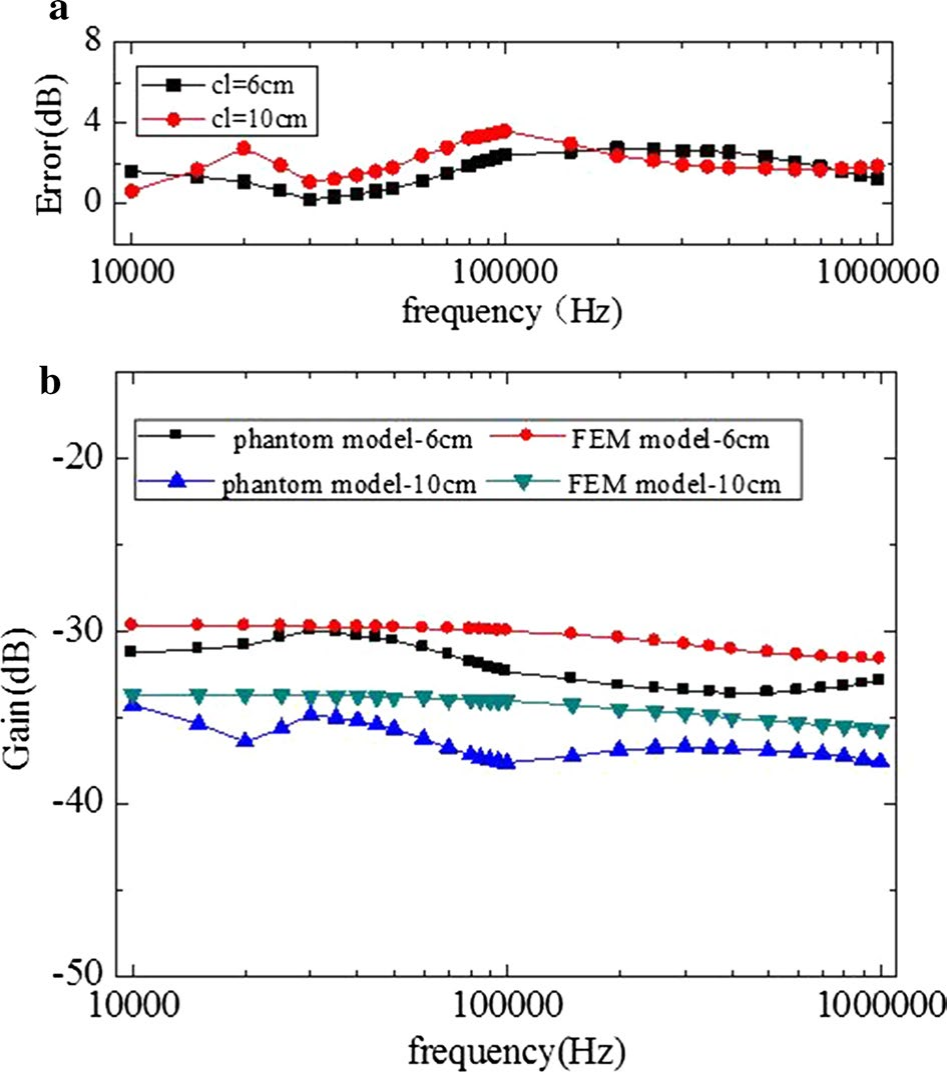
In mode B1, in the direction of the calf, a comparison between implantable FEM model simulation results and phantom model experiment results. **a** Error of the phantom model and FEM model. **b** Results of phantom model experiment and FEM model simulation

**Fig. 12:**
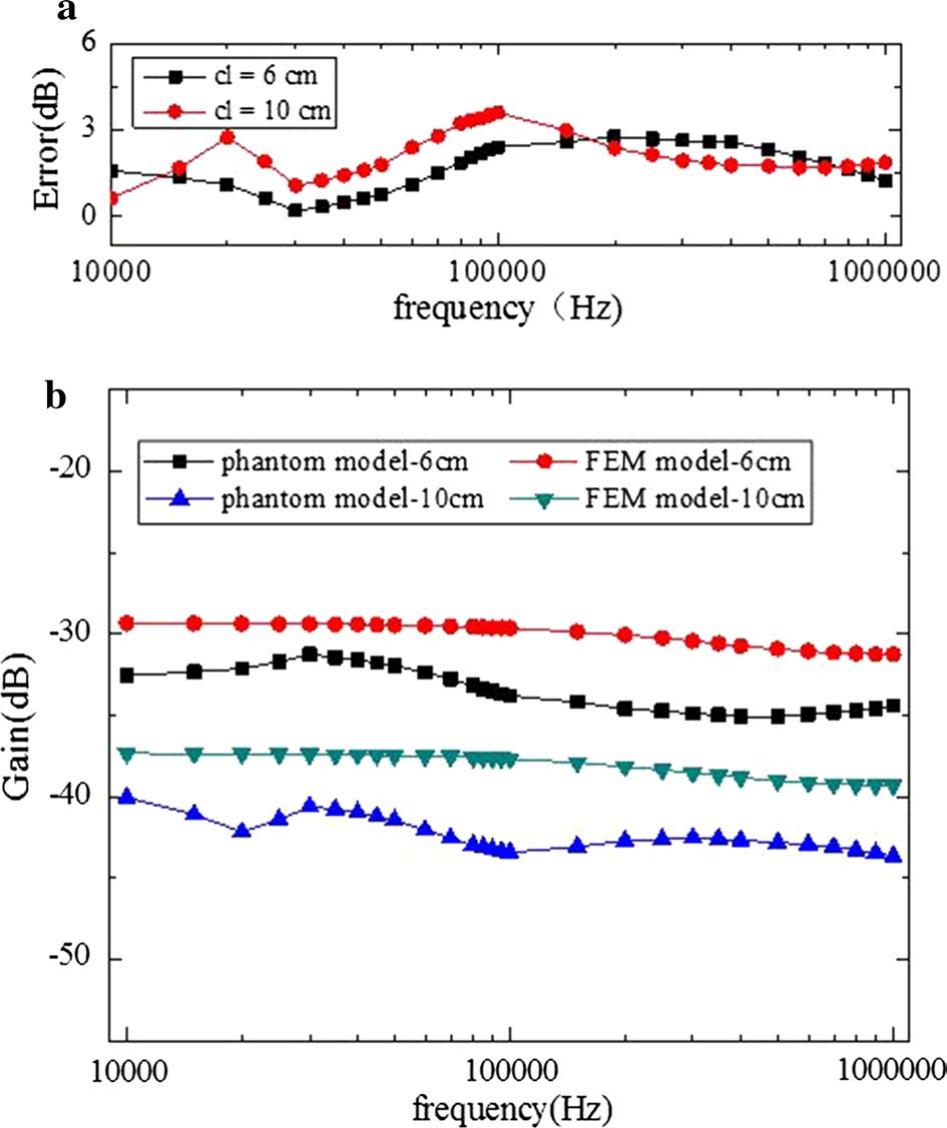
In mode B2, in the direction of the thigh, a comparison between implantable FEM model simulation results and phantom model experiment results. **a** Error of the phantom model and FEM model. **b** Results of phantom model experiment and FEM model simulation

Shown in Figs. 11 and 12, with the implantable receiver electrodes placed at the middle of the leg model (mode B), it could be found that with increasing frequency the signal gain changed little, and the FEM and phantom models had similar channel characteristics. Comparing mode B1 (Fig. 11) with mode B2 (Fig. 12), with transmitter electrodes placed in the direction of thigh or calf, the basic characteristics of the channel were changeless.

**Fig. 13:**
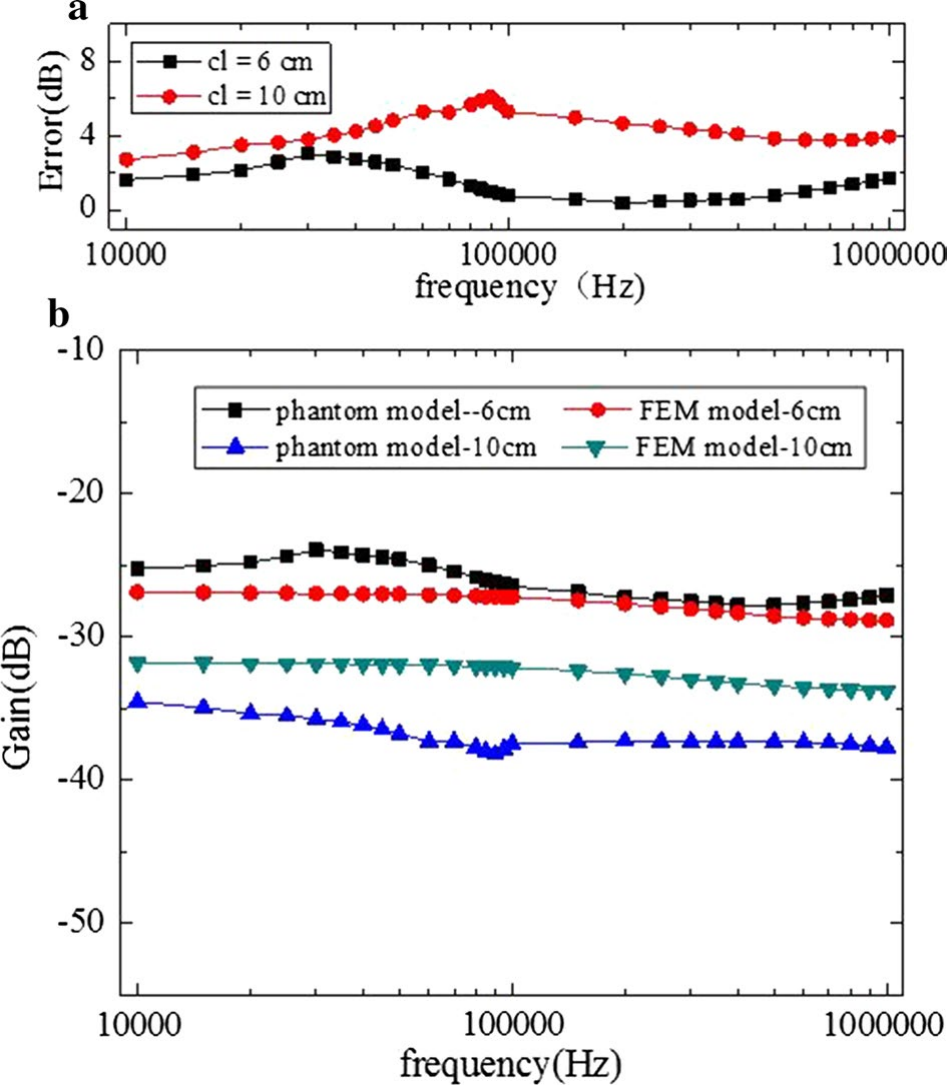
In mode C, a comparison between the implantable FEM model simulation results and the phantom model experiment results. **a** Error of the phantom and FEM models. **b** Results of the phantom model experiment and the FEM model simulation

As shown in Fig. 13, with the implantable receiver electrodes placed in the thigh (mode C), we found that similar to A and B modes the FEM and phantom models had similar channel characteristics, with signal attenuation changeless in the frequencies of 10 kHz to 1 MHz. For the phantom model, when cl = 6 cm, the signal attenuation with the implantable receiver electrodes placed in the calf (mode A) and the thigh (mode C) was approximately 25 dB, which was less than in mode B. When cl = 10 cm, B1 mode and C mode channel characteristic were approximately 35 dB, which were better than that of the other modes. For the FEM model, when cl = 6 cm, the signal attenuation with the implantable receiver electrodes placed in the calf (mode A) and the thigh (mode C) was approximately 27 dB, which was less than that of mode B. When cl = 10 cm, compared with other modes channel characteristics were better in modes B1 and C.

According to the experiment results of the FEM and phantom models, we found that when cl = 6 cm in the frequencies of 10 kHz–1 MHz, with the implantable receiver electrodes placed in the calf (mode A) and the thigh (mode C) the signal attenuation of the phantom model was less than that of the FEM model. In mode B (near the knee), the signal attenuation of the FEM model was less than that of the phantom mode. Finally, when cl = 10 cm, the FEM signal attenuation was less than that of the phantom model in modes A, B, and C.

## Discussion

An implantable FEM model and a phantom model using visible human leg data were created to study surface-to-implant implantable signal transmission characteristics in the frequency range from 10 kHz to 1 MHz at modes A, B, and C in this article. The results showed that the relationship between the voltage attenuation and the frequency of the surface-to-implant signal transmission path was consistent at modes A, B, and C with an increase in frequency, signal attenuation remained basically unchanged. The gain in the fixed attenuation values fluctuated no more than 5 dB, and increasing the channel length increased signal attenuation. It reveals that, in the frequency range of 10 kHz to 1 MHz, the main factors affecting signal transmission characteristics of surface-to-implant was not the frequency, and channel length will be a key parameter to affect the efficiency of power supply of implantable medical devices. In the future, the 10 kHz to 1 MHz frequency range can be used as the working frequency of implantable devices to realize the power supply in body surface. Besides, and in order to get better signal transmission effect and improve the power supply efficiency, the channel lengths of surface-to-implant signal transmission should be shorten as much as possible. The A, B and C modes had the same results, which indicate that the position of the implantable electrode has little effect on the channel characteristics of surface-to-implant signal transmission. The errors of A, B and C was mainly caused by the geometry difference of each tissue layer of the human leg.

The errors of the implantable FEM model and the phantom model were a maximum of 8 dB at modes A, B, and C, with those of the implantable phantom model basically equivalent to those of the implantable FEM model. The cause of the errors of the implantable FEM model and the phantom model was due to the FEM model including four layers of skin, fat, muscle and bone. However, the production process of phantom model ignored the influence of the bone layer, and the skin and fat were synthesized as the skin–fat layer. The phantom model was a two layer model. The phantom experiments carried out in this article were consistent with the results of the FEM model. The Phantom model can be used as an effective supplement to the FEM model in the design and performance test of implantable transceiver, and the research of implantable channel in the future as well.

The study of the surface-to-implant signal transmission characteristics in this article was mainly focused on cl = 6 cm and cl = 10 cm. For the actual BAN (body area network) application scenario, the implantable medical devices were distributed in all parts of the whole human body, and the channel length was likely to be longer than 10 cm. Therefore, further studies on the surface-to-implant signal transmission characteristics of long distance are required. The author’s future work is to establish the FEM model and the phantom model of the whole human body. On the basis of that, we will further explore the surface-to-implant signal transmission characteristics of the longer channel, which provides a theoretical basis for the development of the BAN.

## Conclusions

In this article, we design an implantable FEM model and a phantom model using visible human leg data to study surface-to-implant implantable signal transmission characteristics in the frequency range from 10 kHz to 1 MHz. Comparing the results, with an increase in frequency, surface-to-implant implantable signal attenuation remained basically unchanged, but with an increase in the channel length, signal attenuation increased. It can be concluded that in designing a recharger for implanted medical devices the channel length will be a key parameter. The mutual complementation and authentication of the FEM and phantom models lent veracity and reliability to the implantable signal transmission characteristics studied in this article. The results of this article may benefit the designing of rechargers and implantable transceivers for medical devices.
